# Maximum power in evolution, ecology and economics

**DOI:** 10.1098/rsta.2022.0290

**Published:** 2023-10-02

**Authors:** Charles A. S. Hall, Timothy McWhirter

**Affiliations:** ^1^ Department of Environmental and Forest Biology, SUNY College of Environmental Science and Forestry, Syracuse, NY, USA; ^2^ Montgomery College, Rockville, MD, USA

**Keywords:** maximum power, natural selection, energy, Darwinian

## Abstract

Ludwig Boltzmann suggested that natural selection was fundamentally a struggle among organisms for available energy. Alfred Lotka argued that organisms that capture and use more energy than their competition will have a selective advantage in the evolutionary process, i.e. the Darwinian notion of evolution was based on a fundamental, generalized energy principle. He extended this general principle from the energetics of a single organism or species to the energetics of entire energy pathways through ecosystems. Howard Odum and Richard Pinkerton, building on Lotka, extended this concept to ‘The maximum power principle’ and applied it to many biological and physical systems including human economies. We examine this history and how these ideas relate to concepts from other disciplines including philosophy. But there has been considerable confusion in understanding and applying these concepts which we attempt to resolve while providing various examples from routine life and discussing some unresolved issues.

This article is part of the theme issue ‘Thermodynamics 2.0: Bridging the natural and social sciences (Part 2)’.

## 1. Introduction

The mathematician, physical chemist and statistician Alfred Lotka wrote in 1922 that ‘the two fundamental laws of thermodynamics are, of course, insufficient to determine the course of events in a physical system. They tell us that certain things cannot happen, but they do not tell us what does happen’ [1]. At the beginning of the twentieth century, most scientists understood and accepted that the second law implied that in energy transformations entropy cannot decrease. Many saw this as being inconsistent with the evolution of organized organic systems described in evolutionary biology because living creatures were clearly large bundles of negentropy, i.e. organization, that had not previously existed and as such presented a longstanding paradox that generated severe difficulties in the relations between biology and thermodynamics [2]. Yet the evidence was clear: all kinds of highly organized systems are coming into being continuously—weeds, trees, ecosystems, babies, families, human social systems, hurricanes, planets, solar systems and galaxies. Lotka argued that natural selection leads natural systems to develop in a way that increases the organization of the system to maximize the energy flux through them. He referred to this as the ‘*principle of maximum energy flux*’ [3] and he thought it described the evolution and growth of organized systems—described what does happen. In this sense, he thought of the principle of natural selection ‘functions’ as a fundamental law of thermodynamics [1].

In the 1950s the systems ecologist H. T. Odum and the physicist Richard Pinkerton followed up on Lotka's work in what came to be called the ‘thermodynamic school’ of evolution [4]. They recognized that the first and second laws do not address the *rate* at which energy transformations occur or whether the energy transformations, or flow enhancements, were useful or not. Lotka's *principle of maximum energy flux* depends on Ludwig Boltzmann's idea that in the competitive world in which evolutionary processes take place, organisms can in many cases gain an advantage if they can gather more energy than the competition [5]. Odum and Pinkerton agreed that organisms gain advantage if they can capture energy faster than the competition, preventing the competitors from using a resource first. They went on to demonstrate that the transformations of energy in natural systems are characterized by a unique relation between speed and efficiency such that for many individual processes the most useful energy gained occurred at an intermediate rate that maximized useful power. They eventually came to refer to their version of the principle that guides evolutionary development as the *maximum power principle* (MPP). In physics, energy does not have a time component but power does.

This paper provides a brief discussion of the historical development of the MPP and other related principles. It explains how these principles operate and the evidence supporting them. It illustrates how these principles have been applied to evolutionary theory, ecology, economics and philosophy and discusses some modern continuing uncertainties, research and applications.

## 2. History

### (a) Boltzmann, Lotka, Odum and Pinkerton

Most people today know something about Darwin's concept of natural selection and how it causes species to evolve over time; but many people do not know that Ludwig Boltzmann and Alfred Lotka proposed that natural selection be viewed from a thermodynamic perspective. In 1886 Ludwig Boltzmann wrote ‘The general struggle for existence of animal beings is therefore not a struggle for raw materials—these, for organisms, are air, water and soil, all abundantly available—nor for energy, which exists in plenty in any body in the form of heat, but a struggle for (low) entropy, which becomes available through the transition of energy from the hot sun to the cold earth’ [5]. It is thus rather clear that Boltzmann thinks organisms are competing for ‘low entropy’ sources, or, as Schrödinger put it, ‘negentropy’ [2,5].

Lotka described organisms competing for ‘*available energy*’—(sometimes called *exergy* now; although we use energy here as these authors did.): the organisms that capture and use energy more rapidly and effectively have a selective advantage [3]. As Charles Hall writes, ‘energy is a general resource that can be diverted to whatever contingencies an organism faces, and the maximum accumulation of energy allows maximum reproductive output which is, after all, what natural selection is based on’ [6]. Lotka argued that organisms which capture and use more energy than their competition will have a selective advantage. The first author has been much involved in recent studies of the traditional perspectives of natural selection and their relation to energy (e.g. see Brown *et al*. for a more thorough discussion of the traditional terms of natural selection and their relation to energy [7,8]).

Lotka extended Boltzmann's view of natural selection from species to ecosystems and he describes it using a general principle. After an examination of several cases, Lotka wrote,

‘In every instance considered, natural selection will so operate as to increase the total mass of the organic system, to increase the rate of circulation of matter through the system, and to increase the total energy flux through the system, so long as there is presented an unutilized residue of matter and available energy. This may be expressed by saying that natural selection tends to make the energy flux through the system a maximum, so far as compatible with the constraints to which the system is subject’ [3].

Lotka refers to this as the ‘*principle of maximum energy flux*’ [3]. As we will see, other scientists will go on to develop different aspects of this general principle.

Like Darwin, Lotka believed that his view of natural selection also applied to human beings. He wrote,

‘The question was raised whether, in this, man has been unconsciously fulfilling a law of nature, according to which some physical quantity in the system tends toward a maximum. This is now made to appear probable; and it is found that the physical quantity in question is of the dimensions of power, or energy per unit time … . [3]’

Lotka therefore understood that natural selection was fundamentally a competition among organisms for available energy (and hence the ability to exploit other resources) that generated an evolutionary process that was guided by a *principle of maximum energy flux*.

As mentioned at the outset, initially the second law and biological evolution were viewed as incompatible: how can the organization of life develop over time if entropy increases? In his book *What is Life*, the physicist Erwin Schrödinger considered this question and popularized the idea that living things, like flames (or we add refrigerators), produce entropy at a rate sufficient to compensate for their own internal ordering in a manner consistent with the second law, and that while pockets of negentropy could increase, the entropy of the whole system must also increase. [2]. The Austrian biologist Ludwig von Bertalanffy further developed this approach, arguing that natural systems can spontaneously develop order by extracting then dissipating the potential energy from their environment [9]. The Belgian chemist Ilya Prigogine called these open natural systems *dissipative structures* because they sustain their own organization by dissipating energy gained from their environment [10]. When the dissipative structure of natural systems is analysed as they evolve and grow in the competitive environment of evolutionary processes, the logic that leads to the *principle of maximum energy flux* is reinforced: natural selection will lead dissipative structures to evolve in ways that maximize their ability to gain and then dissipate energy, which requires the flow of energy to be maximized. We are also able to see how Lotka's principle describes the evolution of life in a manner that is consistent with the second law.

Lotka's view inspired the further work by Odum and Pinkerton. Odum had been puzzled by why the efficiency of photosynthesis was so low. In 1955, they wrote a paper, ‘Time's speed regulator’, which provided an explicit analysis of the efficiency and speed of energy transformations [11]. They began by focusing on ‘Atwood's machine’, a pulley with a rope over it attached to two baskets which was at that time a staple of introductory physics laboratories. The machine uses a heavier weight on one side to move a lighter weight up to the top of the system. By adjusting the relative size of the weights, one could examine how differing loading ratios impact the work that could be done per unit of time (friction was assumed negligible). Hall explains what they discovered:

‘One can imagine using elevated rocks to move coal (or gold) from an underground mine to the surface. One can move the coal most rapidly by having a large weight differential in the two baskets—the coal will zip to the top—but not much will be delivered and most of the input energy will end up as heat when the rapidly moving downward basket hits the ground. Alternatively, if the weights are nearly the same, much coal will be delivered — but very slowly. The maximum useful work (maximum useful power) is done when the input energy, the force (weight) of the elevated rocks, is about twice that of the load — *i.e.*, the delivered load — the coal, and about half the input energy is lost as heat’ [6].

Odum & Pinkerton [11] conclude that in order for a system to operate at maximum power, its efficiency should be about 50% of the ideal ‘reversible’ efficiency.

Odum and Pinkerton provide a mathematical derivation for their conclusion based on the concepts developed by Prigogine, Onsager, de Donder, and others [12,13]. Odum & Pinkerton provide a number of examples of systems with variously coupled subsystems with simple but clever diagrams of ecological, biochemical, electrical and economic processes [11]. Odum and Pinkerton believed that studying the selection of maximum rates of flow of benefits at some intermediate efficiency could be used to explain the evolutionary behaviour of all kinds of systems operating in competitive, evolutionary environments, and over the years Odum developed and reported on applications to many different physical, biological, ecological and economic systems.

Many examples of Odum's and Pinkerton's conclusion can be found in everyday life. Hall notes that, ‘…if you want to accelerate on a bicycle (or automobile) you can do the most work (acceleration) in the middle of the appropriate gear range, not at the more efficient lower range or the more rapid upper portion (this can be seen in acceleration graphs in sports car magazines)’ [6]. Thus, Odum and Pinkerton further develop Lotka's *principle of maximum energy flux,* specifically his idea that evolution is guided by ‘the dimensions of power, or energy per unit time…’ [3]. They describe how, in the context of simple energy transfers and transformations, the ‘energy per unit time’ entails a tradeoff between speed and efficiency. Odum later refers to this as the ‘*maximum power principle*’ and he stated it explicitly as follows: ‘Systems that prevail are those with loading adjusted to operate at the peak of the power efficiency curve … . During self-organization, these systems reinforce (choose) pathways with the optimum load and hence optimum efficiency for maximum output’ [14]. He later discussed how such processes would generate the maximum energy-capturing structure possible for a given energy availability and hence powerful ecosystems [15].

One can conclude from Odum's later publications that he believed the MPP was applicable at the scale of organisms, ecosystems and physical systems such as hurricanes and stars [16]. Although Lotka's writings are more vague, he appears to describe the *principle of maximum energy flux* in a similar fashion [3]. The main difference between Lotka's *principle of maximum energy flux* and the MPP is that the latter adds more specificity in what is being selected for by introducing the concept ‘useful energy’ (versus heat), and it provides a mechanism that explains how maximum useful power output is reached through a unique relation between speed and efficiency [11]. Odum's and Pinkerton's use of ‘useful energy’, rather than ‘available energy’, brings both the second law and natural selection into play: evolution cannot operate on low-grade heat; it has to be useful energy that can contribute to survival and reproduction. Both Lotka and Odum are rather ambiguous about whether the mechanisms that generate maximum power, or energy flux, for a whole system operate at the individual species level or at some ‘systems’ level, an issue that is not important for the present discussion, but that is usually resolved in modern science as the collective effects of selection at the level of individuals.

### (b) Maximum power and maximum energy preservation

In the addendum to his 1922 paper which outlines the *principle of maximum energy flux* [3], Lotka considers a principle developed by James Johnstone in a book published the year before which holds that ‘in living processes the increase of entropy is retarded’ along with ‘energy dissipation’. Lotka describes the term *energy flux* as denoting ‘the available energy absorbed by and dissipated within the system per unit of time’ [3]. His *principle of maximum energy flux* therefore describes natural selection leading to the maximization of energy absorption and dissipation and entropy production. Since Johnstone's principle describes ‘energy dissipation’ being ‘retarded’ and Lotka's principle describes it being maximized, Lotka takes some time to consider the possible relation between them.

Lotka proposes two ways that these principles could work together. First, he suggests that Johnstone's argument is not ‘wholly convincing’ because these two principles could work together in a system of ‘coupled transformers'—such as plants and animals—that, as a whole, evolves in the direction outlined by the *principle of maximum energy flux*. The idea is that plants capture energy; animals dissipate it, but animals also provide fertilization and other services that can help the plants grow and hence capture more energy which animals can then dissipate. Lotka writes that in order for Johnstone's argument to be ‘conclusive’ he would have to show that plants and animals work together in a way that, as a system, decreases energy dissipation rather than increasing it; and Lotka notes that Johnstone does not do this.

Second, Lotka proposes that where the supply of energy is limited, the advantage will go to the organism that is most efficient and economical in its internal processes; where the supply of energy is abundant, the advantage will go to the organism that maximizes the dissipative energy flux of the system: so, the two principles can operate in different situations depending on the constraints on natural systems. Lotka concludes by noting that the significance of the phrase ‘compatible with the constraints' remains to be established and he acknowledges that it modifies the meaning of the *principle of maximum energy flux*. Lotka could see that the ways to maximize the energy flux of a system are relative to the ‘constraints’ on that system. Enrico Sciubba suggests in a 2010 paper that Lotka's acknowledgement that the *principle of maximum energy flux* applies in a manner that is ‘compatible with the constraints' on systems is consistent with the random emerging, environment-dependent opportunism’ that defines our modern vision of evolution [17].

Here too Odum and Pinkerton follow Lotka's lead. First, they write that ‘a simple process involving an energy transfer can be considered as a combination of two parts’ [11]: an ‘input and output.’ ‘In one direction’ (i.e. the input), ‘there is the storing of energy, the increase of free energy, and an entropy decrease;’ ‘in the other direction’ (i.e. the output), ‘there is a release of stored energy, a decrease in free energy, and the creation of entropy.’ Johnstone's principle corresponds to the input where entropy is decreased; Lotka's corresponds to the output where entropy is increased. Odum and Pinkerton describe the input and output operating simultaneously and argue that they ‘*perform at an optimum efficiency for maximum (useful) power output*’ (their emphasis). Odum describes this happening at all scales although he also cautioned that it would operate on only one process at a time [18]. He notes that the components of natural systems can specialize in storage and operate at a rate of efficiency that is higher than 50% in order to maximize power for the system as a whole [19]. Fath *et al.* [20] have analysed principles similar to Johnstone's and Lotka's, along with a series of other ‘indicators,’ based on different metrics (energy, emergy, exergy and dissipation) and have concluded that, in the framework of a network analysis, they are all mutually consistent.

Second, Odum and Pinkerton also write that ‘under conditions of limited raw materials as found in many areas of the world, a higher efficiency is the best arrangement…’ [11]. Near the end of his life, Odum wrote a book entitled *A Prosperous Way Down* in which he argued that when the availability of fossil fuels begins to diminish, evolution will begin to select for those societies that develop more efficient modes of production [16]. In his published work throughout his career, Odum often applied the MPP in a manner that was ‘compatible with the constraints' on systems. When we include the phrase ‘compatible with the constraints’ on systems in our definition of the MPP, as Lotka acknowledges, it modifies the meaning of this principle: when a system is constrained by a limited amount of available energy or raw materials, a higher rate of efficiency can be required to maximize power; when available energy and materials are abundant, a lower rate of efficiency can be required to maximize power. Thus, the rate at which power is maximized—time's speed regulator—is relative to the constraints on natural systems.

### (c) Exergy

The science of thermodynamics has advanced in significant ways in recent years through the use of the concept of *exergy* [21], which is defined relative to a system's environment: it is the amount of work a system can perform when it is brought into thermodynamic equilibrium with its environment. It is the useful part of a fuel that can do actual work versus that portion that is turned into heat. When Lotka, Odum and Pinkerton acknowledge that the *principle of maximum energy flux* and the MPP apply to natural systems in a manner that is ‘compatible with the constraints' on systems, they begin to suggest how these principles are the products of a relationship between organisms and systems and their environment. This relationship is further explored in a more detailed fashion by later scientists through the use of the concept of exergy and their work can add greater clarity to the MPP and the MEPP.

There are, however, biological considerations that cannot be overlooked. Boltzmann [5] put biology firmly into the solid format of the physical sciences, which, starting with Carnot, considered the efficiency of a process as being greatly influenced by the difference in temperature between energy source and sink. Boltzman also understood that as various forms of life extracted energy from the environment it was subject to the laws of thermodynamics. However, while it is true that within the context of physics the temperature gradient, e.g. the difference in temperature between source and sink, is important for determining the efficiency of a machine (which an organism is) and is fundamental when starting with the sun, this is superseded at the organismal level by a number of factors: (i) the requirement of life for an operational temperature within which the necessary chemical processes are possible and practical; (ii) the inability of life to alter the external temperature by very much; (iii) natural selection for operating within, and optimizing at, the temperature of its normal habitat and (iv) once photons strike chlorophyll the operational gradient becomes that between highly reduced carbohydrates and oxygen, the ultimate electron sink.

### (d) Maximum empower and its limitations

In the early 1970s, Odum began to recognize that all forms of energy do not have the same ability to do work. He viewed forms of energy in terms of a hierarchy, more diluted forms at the bottom and higher grade forms at the top: the quality of energy is measured by the amount of the lower grade required to develop the higher grade. In order to better standardize the quality of energy, he introduced the concept of *emergy*, i.e. ‘the total amount of available energy of one kind that is directly or indirectly required to make a given product or to support a given flow’ [22]. He used the term *empower* to refer to the flow rate of *emergy*, and he reformulated his MPP as the *maximum empower principle*. This put more distance between his approach and Lotka's, which was outlined in terms of available energy, or *exergy*: it has been argued that *emergy* and *exergy* analyses are not commensurable [23]. The use of *emergy* also eventually came to face challenges. For example, how far back in time do we go to calculate all the energy that went into a product? Since there does not seem to be a clear answer to this question, the accuracy of *emergy* analysis has been questioned [23]. Nevertheless emergy analysis is used routinely and effectively.

### (e) Maximum entropy production

Lotka, Odum and Pinkerton all understood that both the principle of maximum energy flux and the MPP entail the production of entropy. In the twentieth and the twenty-first century, many scientists began to focus on how selection operates to maximize entropy production and they began to use a new principle: the maximum entropy production principle (MEPP) [24–27]. Power is a measure of the dissipation of energy over time; as energy is dissipated, work is done and entropy is produced: so, dissipative power is fundamentally related to the rate of production of entropy. Odum writes that maximum entropy production ‘is another way of referring to maximum power utilization if feedbacks couple the products of power use to power generation’ implying the selection is for ‘useful’ energy capture, not just dissipation [19]. While the MPP and the MEPP are fundamentally related, they reflect different aspects of overall system function and possibly a difference in opinion about exactly what is most important. The MEPP has now been used in several different contemporary scientific disciplines, including physics, chemistry, biology and ecology [27]. Some scientists have suggested that the MEPP could be considered a ‘corollary’ of the second law: it describes how natural systems evolve in a way that is consistent with the second law [27].

In our opinion, the focus on maximum entropy production is missing a critical issue originally put forth by Odum and Pinkerton: in a Darwinian context, it is not the energy captured nor the energy dissipated that is important, rather it is the useful energy that is absorbed by the organism and that is used for survival and reproduction. The high rate of entropy production is selected for, but only incidentally. However, a comprehensive assessment of the relative value of these principles is beyond the scope of this essay.

There is a lineage here: Lotka develops the *principle of maximum energy flux*; Odum and Pinkerton develop the principle further by focusing on the tradeoff between speed and efficiency in coupled systems, useful energy and then explicitly outline the MPP; then some contemporary scientists shift the focus to entropy production and develop the MEPP. The historical evidence suggests that this evolution will continue: our understanding of the relation of energy to evolution will, itself, continue to evolve [28]. Lotka, Odum and Pinkerton were, for the most part, working on their own; now there are a number of scientists in a number of different scientific disciplines using the MEPP in the ‘thermodynamic school’ of evolution. We can see the influence of Lotka, Odum and Pinkerton in this work, as well as the work that will come in the future. Many contemporary scientists acknowledge this influence [24,27].

## 3. Ecology

Although the MPP helps us understand the relation between energy and fitness in the evolutionary process, an important criticism has been raised about it: Hall writes that ‘Odum never really tested it, and, in fact, he said on occasion that, like natural selection, it was extremely difficult if not impossible to test it directly’ [29]. Some scientists that followed Odum have attempted to address this challenge.

Nancy Harris, Charles Hall and Ariel Lugo, for example, did empirical surveys along an elevational gradient in the Luquillo forest in Puerto Rico to test the MPP [30]. Hall writes:

‘We had hypothesized that ecosystems developed the most useful power (net photosynthesis) at intermediate elevations. Gross photosynthesis was maximum at sea level, but there respiration also would be maximum too due to high temperatures. At high elevations photosynthesis is relatively low because of lower sunlight due to cloudiness, but so is respiration because of lower temperature. We developed procedures for measuring photosynthesis and respiration of a column of forest (including all species that were there) using a LI-COR CO_2_ analysis machine and a giant slingshot and rock climbing technology to reach upper portions of the forests…. We found a clear tradeoff between rate and efficiency and a maximum net photosynthesis (useful power) at an intermediate elevation. We wonder whether the environmental conditions at 800 m elevation in the Luquillo Forest represent something of ideal conditions for the balance of photosynthesis and respiration for trees more generally as forest net production also may show a maximum at intermediate latitudes [31,32].’ [6]

The fact that their findings were consistent with their hypothesis provides evidence that the MPP guides the evolution of forests.

Leopold and Langbein provide empirical evidence that tests the MPP in the physical world [33]. They show that streams in tributary networks would disperse their potential energies more quickly if they took more direct routes; instead, what we find in the designs that emerge is that streams in these networks take less direct routes, they meander, and in the process they collect and dissipate more available energy. The structures that develop appear to maximize power. Odum argued that this occurs in physical systems, such as hurricanes and stars [18,34]: structures emerge that enable natural systems to collect and dissipate more available energy.

This conclusion is further supported by a recent study done by Lenton *et al*. [35]. They describe six energy revolutions, three in the history of the earth and three in human history: in earth history, they analyse the origin of anoxygenic photosynthesis, oxygenic photosynthesis and eukaryotic photosynthesis; in human history, they analyse the Paleolithic use of fire, the Neolithic revolution to farming and the Industrial revolution. In each case they attempt to ‘quantify the resulting increase in energy input to the biosphere or to human societies’. Although they do not mention the MPP or the MEPP, their observations are consistent with them. Their research supports these principles in a historical fashion that is similar to the way the fossil record supports the concept of punctuated equilibrium [36]. Their research also illustrates a fundamental parallel between ecological systems and human social systems.

To carry this parallel further, the Russian Biophysicist Aleksandr Zotin writes that when human beings began to expend energy outside their bodies through the use of fire and machines, it enabled them to surpass the biological limits of their bodies and further maximize their ability to manifest power and produce entropy [27]. When we consider the different ways human beings have transformed energy outside their bodies historically, we can see that the thesis developed by Lenton *et al.* can be extended to the first three industrial revolutions. Above is a partial, chronological list of the advancements in heat engine technology provided by Earl Cook (table 1) [37].

**Table:** 

machine	date	horsepower
man pushing a lever	3000 BC	0.05
Vitruvian water mill	50 BC	3
post windmill	AD 1400	8
Watt's steam engine (land)	AD 1800	40
marine steam engine	AD 1900	8000
coal-fired steam power plant	AD 1973	1 465 000
nuclear power plant	AD 1970	1 520 000

This list illustrates that the power of these machines continues to go up over time and there are nonlinear increases that correspond to each of the first three industrial revolutions: 1st 1760–1840, 2nd 1870–1914 and 3rd 1947–2009. This point will be further supported in the next section with a discussion of how the industrial revolutions coincide with the discovery and increasing use of fossil fuels. Together, the research by Lenton *et al.*, Zotin and Cook provide powerful historical evidence that ecological systems and human social systems have evolved in a manner that is consistent with the MPP and the MEPP. Thus, it seems that this is an important insight into how natural and human selective processes work, and is a fertile area for continued study.

## 4. Economics

### (a) Energy and neoclassical economics

Odum could see the MPP at work in contemporary human societies: structures emerge that collect and dissipate more fossil fuels to the extent that they are available. Hall writes that Odum ‘felt that societies or groups that chose not to do this could be more efficient but would be overtaken by systems that continued to focus on increasing their rate of exploitation of fossil fuels, at least while fuels were abundant’ [6]. Consequently, when fossil fuels were abundant in the 1960s, Odum believed that the US should use them first, or at least ‘not take themselves out of the race to use fossil fuels’, despite the fact that many people at that time, including his own graduate students, believed that we needed to reduce industrialization to reduce pollution [29].

More and more scientists—starting more than 100 years ago with Ukrainian Physician Serge Podolinski, the chemist Frederick Soddy and the sociologist Frederick Cottrell—have recognized that the key to understanding human history and economics is understanding how humans have used energy. Hall provides a brief description of the role energy played in the creation of contemporary industrial societies,

‘The principal energy sources in antiquity were all derived directly from the sun: human and animal muscle power, wood, flowing water, and wind. About 300 years ago, the industrial revolution began. It brought an exponential increase in the energy available to humans to do economic work. This revolution began with stationary wind-powered and water-powered technologies, which were subsequently supplemented and replaced by fossil hydrocarbons (fossil meaning old): coal in the nineteenth century, oil in the twentieth century, and now, increasingly, natural gas. The global use of hydrocarbons for fuel by humans has increased nearly 800-fold since 1750 and about 12-fold in the twentieth century. The enormous expansion of the human population and the economies of most nations in the past 100 years have been facilitated by a commensurate expansion in the use of fossil fuels. Perhaps the industrial revolution should be renamed ‘the hydrocarbon revolution’ [6].

The MPP provides an explanation for the nature of the evolution of contemporary socio-economic systems. It describes the evolution of these systems as being driven by physical laws. Odum did not believe that human actions were determined; he thought humans were free to choose what they do, but evolutionary processes would ensure that over time those humans that behave in a manner that is consistent with the MPP would have a selective advantage. Therefore, as Hall describes, ‘human behaviors are both driven by and constrained by energetic principles whether or not humans are aware of it’ [6]. Like evolution itself, understanding this aspect of the MPP is not necessarily a pretty thing. The brutal wars and the extraordinary forms of exploitation of environments and other human beings we find in our past appear to have been selected for. Is the MPP at work now? Is it necessary to divert much of the United States' remaining fossil fuels to arm Ukraine in military competition with Russia? Is the competition among nations to use the world's resources undermining our ability to address global climate change? Or in the longer run does natural selection reward relatively ‘virtuous’ behaviour through some kind of larger scale evolution?

Addressing these questions in the depth that is appropriate to them would take us way beyond the scope of this essay; but we can here focus attention on the evidence offered so far that supports Lotka's suggestion that ‘man has been unconsciously fulfilling a law of nature, according to which some physical quantity in the system tends toward a maximum’ [3]. The evolution of the use of energy in much of human life has proceeded in a manner that appears to be at least consistent with the MPP, and it is difficult to overstate the nature of the impact this has had on contemporary socio-economic systems.

**Figure 1.**
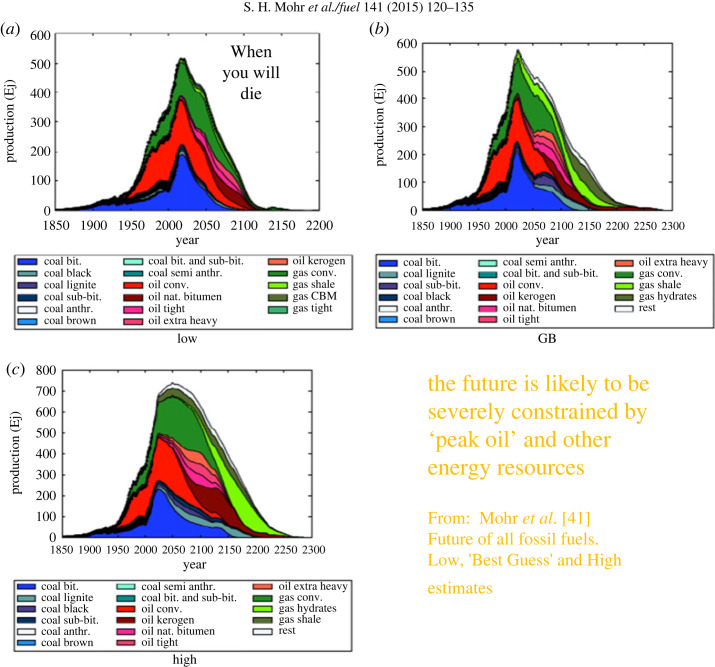
Past production of fossil fuels and three projections of future availability: (*a*) Low estimate (*b*) best-guess, and (*c*) high (maximum) estimate [42]. Similar estimates are available from Maggio and Cacciola [43] and Laherrere *et al*. [44]. All three estimates show a peak of all fossil fuels within at most the next 10–35 years.

Modern neoclassical economics, for the most part, ignores the relation between energy use and economic activity and therefore provides an incomplete picture. It is fundamentally based on the concept of ‘consumer sovereignty’: the idea that economic value is determined by the subjective judgements of consumers and economic markets share this information in an undistorted manner. Many disagree with this view (Hall *et al*. [38], Sekera [39], Leontief [40], Tao *et al*. [41]). Contemporary neoclassical economists and politicians generally believe that we should attempt to continually foster economic growth because this is the only way to enhance human well-being. This view of economics, which emerged during a unique period where there was an unprecedented increase in our use of fossil fuels, might seem to have been consistent with the MPP but does not consider the idea that resource shortages might be important. As we approach the projected peak of the availability of fossil fuels, this failure becomes more and more important (figure 1). Meanwhile, the depletion of our energy resources continues relentlessly [44].

### (b) Biophysical economics

Economics has not always been viewed in this neoclassical manner. In the eighteenth century, the French physiocrats viewed land and agriculture as the source of wealth. In the 1960s, scientists and economists began to focus on the joint application of exergy analysis and engineering economics; in Europe, the approach was called exergo-economics; in the US it went by the name thermo-economics [21]. In the 1980s, an approach emerged called BioPhysical economics, which takes into consideration the fact that economic systems, like all natural systems, are governed by thermodynamic laws and principles, including the MPP [45,46]. Like exergo-economics and thermo-economics, BioPhysical economics seeks to better understand economic processes by acknowledging the central role played by energy. In the process, it attempts to address many of the problems with neoclassical economic theory. We briefly summarize two aspects of this view below.

First, BioPhysical economics considers the implications of the fact that there is a limited supply of fossil fuels on the planet and we are quickly reaching the peak of their availability ([46]; see also www.bpeinstitute.org); there is also a limited supply of raw materials. It acknowledges that the process of converting economies to renewable forms of energy is a long one, fraught with all kinds of challenges and limitations. Consequently, it questions the fundamental assumption in neoclassical economics that continual economic growth is possible and should be the goal of all economic activity. Hall writes, ‘BioPhysical economics considers and encourages the possibility that humans are capable of achieving happiness by means other than the acquisition of ever-increasing quantities of material goods—goods that cannot be produced with declining resources’ [6]. It maintains that the focus in economics should be on living well within the limits of nature. As mentioned (on p. 9), Odum agreed with this view later in his life [16]. Hall writes that he could see that we were nearing the peak of the availability of fossil fuels and ‘that we should redirect our efforts away from luxury consumption and blind competition toward investments into low energy consuming infrastructure, education, and even birth control’ [29]. He believed on the other side of this peak there would be selection for societies that adapted better to this new environment—these new constraints. He understood that the MPP applies in a way that is ‘compatible with the constraints' on natural and economic systems [16].

**Figure 2.**
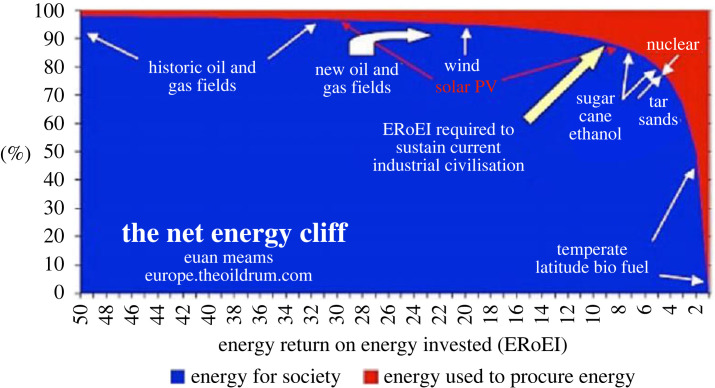
The ‘net energy cliff’. As EROI declines from high values to lower values, it makes little difference to society, as the net energy delivered does not change much. But below an EROI of 10 : 1 any declines make a large difference on the amount delivered (darker colour) (Courtesy of Euan Mearns).

Second, BioPhysical economics provides guidelines for the assessment of alternative sources of energy. Here the concept of *energy return on investment* (EROI) stands out as an important metric. It is the ratio of the amount of energy delivered from a particular energy resource to the amount of energy used to obtain that energy resource. This concept is presently used to evaluate all kinds of energy sources [6,47]. It is defined in terms of available energy. Other scientists have gone on to formulate the concept more explicitly in terms of exergy: Exergy Return on Exergy Invested [48,49]. EROI and related concepts will become more important going forward. Heinberg and Fridley estimate that if we are to shift to a 100% solar society over the next several decades, the financial investments required will need to be 20 times all of our renewable energy investments up to the present in each of 20 succeeding years [50]. Imagine for a moment what the consequences would be if a society made this kind of historic investment in the wrong energy resource, such as corn-based ethanol. Would it ever recover? This highlights the strategic value of using EROI and related concepts to guide our future investments; see figure 2.

Neoclassical economics dominated the study of economics in the twentieth century, during a period where our use of fossil fuels was increasing at an unprecedented and ultimately unsustainable rate. But those days are over. It is now the economics of the past. BioPhysical economics provides a more comprehensive view of economic processes that is in a better position to help us manage our way through the challenges we face now and in the future.

## 5. Maximum power, science and philosophy

The MPP has roots in philosophy. Thirty-six years before Lotka argued that evolution was guided by his *principle of maximum energy flux*, the nineteenth-century German philosopher Friedrich Nietzsche argued that evolution was guided by a ‘*will to power*,’ which leads natural systems to develop in ways that increase their power [51]. His work was influenced by a number of eighteenth- and nineteenth-century natural scientists: Roger Boscovich, Jean-Marie Guyau, William Rolph, Julius Mayer and Maximilian Drossbach. Drossbach writes, ‘we only have a proper understanding of force if we recognize it as the striving for expansion [*Streben nach Entfaltung*]’ [52]. Nietzsche agreed.

Nietzsche used his concept of the will to power to take a unique, naturalistic approach to his critique of philosophy. It provided a foundation for a thermodynamic framework for the growth and flourishing of life that he used to *describe* how different forms of behaviour and knowledge either fostered or undermined this growth. He did not therefore criticize moral values for being immoral or forms of knowledge for being untrue, as most philosophers attempted to do; he attempted to view humanity from a perspective ‘hardened in the discipline of science’ [51,53]. He viewed his famous critique of morality as a ‘science of morals.’ He saw himself as a cultural ‘physician’ that cuts away that which undermines growth and encourages the use of ‘natural’ or ‘healthy’ moralities that remove ‘hostile’ elements ‘on the path’ of the growth of life [54].

The best example of a twentieth-century cultural physician would have to be H. T. Odum. In his book *Environment, Power, and Society* [55], Odum evaluates moral values, religions, and political systems from the perspective of a thermodynamic framework of growth based on the MPP. The similarities in Odum's and Nietzsche's perspectives are quite extraordinary; but we can also learn a great deal from the differences in their analyses. They have very different views about the value of democracy, religion and war, among other things. Nietzsche thinks democracy, religion and peace undermined the growth of life. Odum argues from the perspective of an evolutionist that democracy provides valuable information and choices to leaders that can foster growth; certain forms of religion can foster growth and war can foster growth at certain stages, but eventually it can come to undermine the growth of mature social systems. If we take into consideration the unique perspective they share, these important differences make sense. They are not analysing social systems from the perspective of some ahistorical conception of truth; they are analysing them from a scientific perspective that evolves over time. The treatments prescribed by twentieth-century physicians are very different from those prescribed by nineteenth-century physicians. It is certain that there will be enormous changes in our energy regimes over the next half-century. If the changes we make in our economic and political philosophies and structures are not consistent with good science, our efforts will fail. A critical issue is that if, and as, EROI continues to decline, inflation is inevitable. People tend to blame inflation on governments, which will make polities increasingly ungovernable -- just as we need good government.

## 6. Conclusion

The *principle of maximum energy flux* has come a long way over the past 100 years. Alfred Lotka argued that it guided the evolution of organic and inorganic systems, fundamentally revising Darwin's theory of evolution. Odum and Pinkerton further developed the principle, arguing that the MPP depended on a tradeoff between speed and efficiency that maximizes useful power. Odum went on to apply the MPP to physical, electrical, biochemical, biological, ecological and economic systems. Others have followed in his footsteps. Now the related MEPP is used in several different scientific disciplines and there is compelling empirical evidence that supports it.

The application of the MPP and other related principles to human social systems raises a number of questions that clearly deserve more attention. So far, the application of these principles has led to a thorough critique of neoclassical economics and the emergence of a new approach, called BioPhysical economics [45,46]. This new view acknowledges the central role energy plays in all economic processes and realistically considers the economic implications of the limited supply of fossil fuels that have propelled the growth of economies over the last two centuries. It provides conceptual tools, such as EROI, that can help guide economies through the transition to renewable forms of energy. It also illustrates how to close the gap between the natural and the social sciences. In philosophy, the MPP opens up the possibility of a unique naturalistic approach to philosophical questions, which would close the gap between the sciences and the humanities. In the work of Nietzsche, Lotka, Odum and many who have followed them, we find this provocative assertion—this world and all of us in it are the products of a fundamental tendency to maximize power.

## Data Availability

This article has no additional data.

## References

[1] Lotka A. 1922 Natural selection as a physical principle. Proc. Natl Acad. Sci. USA **8**, 151-154. (10.1073/pnas.8.6.151) 16576643 PMC1085053

[2] Schrödinger E. 1945 What an organism feeds upon is negative entropy In What is life? The physical aspect of the living cell (ed. E Schroedinger). New York, NY: Macmillan.

[3] Lotka A. 1922 Contribution to the energetics of evolution. Proc. Natl Acad. Sci. USA **8**, 147-151. (10.1073/pnas.8.6.147) 16576642 PMC1085052

[4] Fry I. 1995 Evolution in thermodynamic perspective: a historical and philosophical angle. Zygon **30**, 227-248. (10.1111/j.1467-9744.1995.tb00067.x)

[5] Boltzmann L. 1886 The second law of thermodynamics. In Ludwig Boltzmann: theoretical physics and philosophical problems: selected writings (ed. B McGinness), pp. 14-32. Dordrecht, The Netherlands: D. Reidel, 1974.

[6] Hall CAS. 2017 Energy return of investment: a unifying principle for biology, economic, and sustainability. Berlin, Germany: Springer.

[7] Brown J, Burger R, Hou C, Hall CAS. 2022 The pace of life: metabolic energy, biological time, and life history. Integr. Comp. Biol. **62**, 1-13. (10.1093/icb/icac120) 35903994

[8] Brown J, Hall C, Burger R, Hou C. In preparation. Physics, physiology and fitness: What does natural selection maximize?

[9] Bertalanffy L. 1952 Problems of life. London, UK: C. A. Watts & Co.

[10] Prigogine I. 1961 Thermodynamics of irreversible processes, 2nd edn. New York, NY: Interscience Publishers.

[11] Odum HT, Pinkerton R. 1955 Time's speed regulator: the optimum efficiency for maximum power output in physical and biological systems. Am. Sci. **43/2**, 331-343.

[12] De Groot SR. 1951 Thermodynamics of irreversible processes. New York, NY: Interscience Publishers.

[13] Denbigh KG. 1951 Thermodynamics of the steady state. London, UK: Methuen.

[14] Odum HT. 2007 Environment, power, and society for the 21th century. New York, NY: Columbia University Press.

[15] Odum HT. 1994 Ecological and general systems. Boulder, CO: University Press of Colorado.

[16] Odum HT, Odum EC. 2001 A prosperous Way down. Boulder: University Press of Colorado. Kindle version. Also C.H. personal conversation with HTO.

[17] Sciubba E. 2010 What did Lotka really say? A critical reassessment of the ‘maximum power principle. Ecol. Model. **222**, 1348. (10.1016/j.ecolmodel.2011.02.002)

[18] Odum HT. 1995 Self-organization and maximum empower. In Maximum power: the ideas and applications of H. T. Odum (ed. CAS Hall). Boulder, CO: University Press of Colorado.

[19] Odum HT. 1983 Maximum power and efficiency: a rebuttal. Ecol. Model. **20**, 71-82. 73. (10.1016/0304-3800(83)90032-7)

[20] Fath BD, Patten BC, Choi JS. 2001 Complementarity of ecological goal functions. J. Theor. Biol. **208**, 493-506. (10.1006/jtbi.2000.2234) 11222052

[21] Sciubba E, Wall G. 2007 A brief commented history of exergy from the beginnings to 2004. Int. J. Thermodyn. **10**, 1-26.

[22] Odum HT. 1996 Environmental accounting. Emergy and environmental decision making. New York, NY: J. Wiley and Sons.

[23] Sciubba E. 2009 Why emergy- and exergy analyses are non-commensurable methods of system analysis. Int. J. Exergy **6**, 523-549. (10.1504/IJEX.2009.026676)

[24] Kleidon A, Lorenz RD. 2005 NET and the production of entropy: life, earth and beyond. New York, NY: Springer.

[25] Kleidon A, Dyke J. 2010 The maximum entropy production principle: its theoretical foundations and applications to the earth system. Entropy **12/3**, 613-630. (10.3390/e12030613)

[26] Swenson R. 2009 The fourth law of thermodynamics. Chemistry **8**, 333-339.

[27] Martyushev LM, Seleznev VD. 2006 Maximum entropy production principle in physics, chemistry and biology. Phys. Rep. **406**, 1-45. (10.1016/j.physrep.2005.12.001)

[28] Brown JH, Hall AS, Sibly RM. 2018 Equal Fitness paradigm explained by a trade-off between generation time and energy production rate. Nat. Ecol. Evol. **2**, 262-268. (10.1038/s41559-017-0430-1) 29311701

[29] Hall CAS. 2004 The continuing importance of maximum power. Ecol. Model. **178**, 107-113. (10.1016/j.ecolmodel.2004.03.003)

[30] Harris NL, Hall CAS, Lugo AE. 2013 A test of the maximum power hypothesis along an elevational gradient in the Luquillo Mountains of Puerto Rico. Ecol. Bullet. **54**, 233-243.

[31] Gillman LN, Wright SD, Cusens J, McBride PD, Malhi Y, Whittaker RJ. 2015 Latitude, productivity and species richness. Glob. Ecol. Biogeogr. **24**, 107-117. (10.1111/geb.12245)

[32] Huston MA, Wolverton S. 2009 The global distribution of net primary production: resolving the paradox. Ecol. Monogr. **79**, 343-377. (10.1890/08-0588.1)

[33] Langbein WA, Leopold B. 1966* Theory of minimum variance. Physiographic and Hydraulic studies of rivers. Geological Survey Professional Paper 422-H.* Washington, DC: USGS.

[34] Odum HT. 1994 Ecological and general systems: an introduction to systems ecology. Niwot, CO: University Press of Colorado.

[35] Lenton TM, Pichler P, Weiz H. 2016 Revolutions in energy input and material cycling in Earth history and human history. Earth Syst. Dyn. **7**, 353-370. (10.5194/esd-7-353-2016)

[36] Eldredge N, Gould SJ. 1972 Punctuated equilibria: an alternative to phyletic gradualism. In Models in paleobiology (ed. TJM Schopf), pp. 82-115. San Francisco, CA: Freeman Cooper.

[37] Cook E. 1976 Man, energy, society. San Francisco, CA: W. H. Freeman.

[38] Hall CAS, Lindenberger D, Kummel R, Kroeger T, Eichhorn W. 2001 The need to reintegrate the natural sciences with economics. Bioscience **51**, 663-673. (10.1641/0006-3568(2001)051[0663:TNTRTN]2.0.CO;2)

[39] Sekera JA. 2016 The public economy in crisis: a call for a new public economics. New York, NY: Springer Briefs in Economics.

[40] Leontief WW. 1982 Academic economics. Science **217**, 104-107. (10.1126/science.217.4555.104) 17770240

[41] Tao Y, Wu X, Zhou T, Yan W, Huang Y, Yu H, Mondal B, Yakovenko V. 2019 Exponential structure of income inequality: evidence from 67 countries. J. Econ. Interact. Coord. **14**, 345-376. (10.1007/s11403-017-0211-6)

[42] Mohr SH, Wang J, Ellem G, Ward J, Giurco D. 2015 Projection of world fossil fuels by country. Fuel **1**, 120-135. (10.1016/j.fuel.2014.10.030)

[43] Maggio G, Cacciola G. 2012 When will oil, natural gas, and coal peak? Fuel **31**, 111-123. (10.1016/j.fuel.2012.03.021)

[44] Laherrere J, Hall CAS, Bentley R. 2022 How much oil remains for the world to produce? Comparing assessment methods, and separating fact from fiction. Curr. Res. Environ. Sustain. **4**, 100174. (10.1016/j.crsust.2022.100174)

[45] Cleveland CJ, Costanza R, Hall CAS, Kaufman R. 1984 Energy and the U. S. economy: a biophysical perspective. Science **225**, 890-897. (10.1126/science.225.4665.890) 17779848

[46] Hall CAS, Klitgaard K. 2017 Energy and the wealth of nations. New York, NY: Springer.

[47] Oosterom JP, Hall CAS. 2022 Enhancing the evaluation of Energy Investments by supplementing traditional discounted cash flow with Energy Return on Investment analysis. Energy Policy **168**, 112953. (10.1016/j.enpol.2022.112953)

[48] Farajzadeh F, Lomans BP, Hajibeygi H, Bruining J. 2022 Exergy return on exergy investment and CO_2_ intensity of the underground biomethanation process. ACS Sustai. Chem. Eng. **10**, 10 318-10 326. (10.1021/acssuschemeng.2c02931)

[49] Hassan AM, Ayoub M, Eissa M, Musa T, Bruining H, Farajzadeh R. 2019 Exergy return on exergy investment analysis of natural-polymer (Guar-Arabic gum) enhanced oil recovery process. Energy **181**, 162-172. (10.1016/j.energy.2019.05.137)

[50] Heinberg R, Fridley D. 2016 Our renewable future: laying the path for one hundred percent clean energy. Washington, WA: DC: Island Press.

[51] Nietzsche F. 1886/1989 *Beyond Good and Evil* [*Jenseits von Gut und Böse. Vorspiel einer Philosophie der Zukunft*]. Translated by Walter Kaufmann. New York, NY: Vintage.

[52] Drossbach M. 1884 Über die scheinbaren und die wirklichen Ursachen des Geschehens in der Welt. Halle/Saale, Germany: Pfeffer.

[53] McWhirter T. 2012 Nietzsche's naturalistic metaethics: in defense of privilege. Phil. Study **2/2**, 92-102.

[54] Nietzsche F. 1888/1968 Twilight of the idols, in The Portable Nietzsche, trans. and ed. W. Kaufmann. New York, NY: Viking Penguin.

[55] Odum HT. 1971 Environment, power, and society. New York, NY: John Wiley & Sons.

